# Understanding the *Modus Operandi* of Class II KNOX Transcription Factors in Secondary Cell Wall Biosynthesis

**DOI:** 10.3390/plants11040493

**Published:** 2022-02-11

**Authors:** Akula Nookaraju, Shashank K. Pandey, Yogesh K. Ahlawat, Chandrashekhar P. Joshi

**Affiliations:** 1 Department of Biological Sciences, Michigan Technological University, Houghton, MI 49931, USA; nookarajuakula@gmail.com (A.N.); shashank.pandey@slu.se (S.K.P.); yogesh.ahlawat@ufl.edu (Y.K.A.); 2 Kaveri Seed Company Limited, Secunderabad 500003, Telangana, India; 3 Umeå Plant Science Centre, Department of Forest Genetics and Plant Physiology, Swedish University of Agricultural Sciences, SE-901 87 Umeå, Sweden; 4 Department of Horticultural Sciences, University of Florida, Gainesville, FL 32611, USA

**Keywords:** bioethanol, KNOX II transcription factors, saccharification, secondary cell walls, xylan, xylem and fiber development

## Abstract

Lignocellulosic biomass from the secondary cell walls of plants has a veritable potential to provide some of the most appropriate raw materials for producing second-generation biofuels. Therefore, we must first understand how plants synthesize these complex secondary cell walls that consist of cellulose, hemicellulose, and lignin in order to deconstruct them later on into simple sugars to produce bioethanol via fermentation. *Knotted-like* homeobox (*KNOX*) genes encode homeodomain-containing transcription factors (TFs) that modulate various important developmental processes in plants. While *Class I KNOX TF* genes are mainly expressed in the shoot apical meristems of both monocot and eudicot plants and are involved in meristem maintenance and/or formation, *Class II KNOX* *TF* genes exhibit diverse expression patterns and their precise functions have mostly remained unknown, until recently. The expression patterns of *Class II KNOX TF* genes in *Arabidopsis*, namely *KNAT3, KNAT4, KNAT5,* and *KNAT7*, suggest that TFs encoded by at least some of these genes, such as *KNAT7* and *KNAT3*, may play a significant role in secondary cell wall formation. Specifically, the expression of the *KNAT7* gene is regulated by upstream TFs, such as SND1 and MYB46, while KNAT7 interacts with other cell wall proteins, such as KNAT3, MYB75, OFPs, and BLHs, to regulate secondary cell wall formation. Moreover, KNAT7 directly regulates the expression of some xylan synthesis genes. In this review, we summarize the current mechanistic understanding of the roles of Class II KNOX TFs in secondary cell wall formation. Recent success with the genetic manipulation of Class II KNOX TFs suggests that this may be one of the biotechnological strategies to improve plant feedstocks for bioethanol production.

## 1. Introduction

Increasing global demand for petroleum-based transportation fuels has created an imperative need for the search and development of alternative, sustainable, and renewable sources of bioenergy. First-generation biofuels are produced from starch and sugars that come from agricultural crops. Such applications are often associated with direct competition with food resources and are with some important ethical, ecological, and societal issues [1]. Second-generation biofuels are produced from lignocellulosic biomass derived from the secondary cell walls (SCWs) of plants that can be used to produce alternative transportation fuels, such as bioethanol. Despite its abundance, only a small portion of lignocellulosic biomass is presently used for bioethanol production, owing to its recalcitrance for the bioconversion to bioethanol. Additionally, converting woody biomass into fermentable sugars still requires high input technologies involving extensive pre-treatments and expensive enzymes [2,3]. Hence, a clear understanding of the plant metabolic processes influencing SCW properties will assist in improving plant feedstocks for bioethanol production [3].

Second-generation biofuels have become an important component of the global bioenergy agenda [2]. The use of plant biomass for the production of bioethanol has an enormous potential to revolutionize the global bioenergy outlook. Scientists have discovered novel ways of improving lignocellulosic biomass production in bioenergy crops and trees, such as switchgrass and poplars, that could be used for efficient biofuel production [4,5,6]. The SCW is mainly composed of cellulose, hemicelluloses, and lignin; one of the primary roles of SCW is to confer mechanical strength to plant tissues [6]. However, the main carbohydrate components of SCWs—cellulose and hemicellulose—can be deconstructed into simple sugars (saccharification), and these sugars can subsequently be fermented to produce bioethanol [3]. During the last two decades, several studies have been initiated to understand and modify the biosynthesis of cellulose, hemicellulose, and lignin through the manipulation of genes involved in these pathways [6]. In addition, several members of the NAC, MYB, and KNOX transcription factor (TF) families have been studied to elucidate their regulatory roles in SCW biosynthesis [7,8,9,10]. These TFs function by regulating the SCW biosynthetic genes that encode cellulose synthases (CesAs), xylan synthases, and lignin biosynthetic pathway enzymes. One of the *Class II KNOTTED1-like homeodomain* (*KNOX*) genes, *KNAT7*, has recently gained attention for its potential role in the transcriptional network regulating SCW biosynthesis [11,12,13,14,15,16,17]. This comprehensive review focuses on the recent developments in our understanding of the transcriptional networks involving Class II KNOX TFs in the regulation of SCW biosynthesis.

### 1.1. KNOX Genes and Encoded KNOX Proteins in Plants

**Figure 1:**
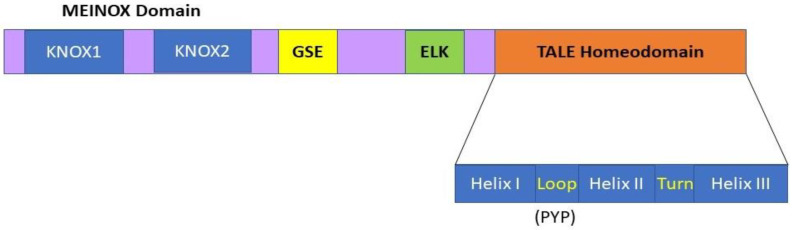
KNOX protein domain organization comprising MEINOX, ELK, and the TALE homeodomain (HD). The TALE homeodomain consists of three α-helices which comprise a helix-turn-helix type DNA binding motif, and contains three extra residues (PYP) in the loop between the first and second helices as compared to typical HDs. The MEINOX domain is present at the N terminus of the KNOX proteins, and it functions during protein–protein interactions. This MEINOX domain in plants is made of two smaller domains, KNOX1 and KNOX2, separated by a linker sequence. The ELK domain has been suggested to function as a nuclear localization signal and be involved in protein–protein interactions. The relatively small and less well-conserved amino acid motif located between the MEINOX and ELK domains is called the GSE domain; its function is not well understood.

The *KNOX* genes are members of one of the ancestral gene families involved in the transition of plants from an aquatic to a terrestrial habitat during evolution [18]. *KNOX* genes encode homeodomain (HD)-containing TFs involved in various developmental processes. Typical HD proteins contain 60 amino acids, while the HD of KNOX proteins contains a highly conserved 63-amino acid stretch consisting of three ∝-helices that form a helix-turn-helix-type DNA binding motif [19] (Figure 1). Due to the presence of three extra amino acids between the first and second helices, all KNOX TF proteins are included in the TALE (three amino acid loop extension) superclass, the members of which are evolutionarily conserved from single-cell algae to higher plants [20]. The sequence immediately upstream of the HD, the ELK domain, has been suggested to function as a nuclear localization signal and be involved in protein–protein interactions [20]. In addition to the HD and ELK domains, a stretch of 100 amino acids located at the N terminus of almost all KNOX proteins, the MEINOX domain, also functions in protein–protein interactions [20]. This MEINOX domain in plants consists of two smaller domains, KNOX1 and KNOX2, separated by a poorly conserved linker sequence (Figure 1).

Plant *KNOX* genes are divided into three subclasses based on their sequence similarity within the HD encoding regions, intron positions, expression patterns, and phylogenetic analysis [21,22,23,24]. *Class I KNOX* genes are similar to the *knotted1* gene of maize [25] and are mainly expressed in the shoot apical meristems (SAMs) of both monocot and eudicot plants. The *Class I KNOX* genes *STM*, *KNAT1*/*BP*, *KNAT2*, and *KNAT6* in *Arabidopsis* play an important role in the transcriptional regulation of meristem development, leaf shape control, and hormone homeostasis [26]. Loss-of-function mutations in these genes affect meristem maintenance and/or formation [27]. The only member of *Class III KNOX* gene, *KNATM*, is involved in the regulation of leaf polarity, leaf shape, and compound leaf development [28]. Four *Class II KNOX* genes (*KNAT3, KNAT4, KNAT5,* and *KNAT7*) in *Arabidopsis* form a separate monophyletic group and have several orthologues in higher plant genomes with few known functions [23,24,29]. Interestingly, *Class II KNOX* genes have been suggested to regulate the haploid-to-diploid morphological transition in land plants [18]. The first plant homeobox gene was discovered over 25 years ago; however, we only recently began to decipher the roles of *Class II KNOX* genes in higher plant growth and development. This review focuses on the functions of *Class II KNOX* genes and their encoded proteins in higher plants. 

### 1.2. The Expression Patterns of Class II KNOX Genes in Plants Provide Some Clues about Their Functionality in SCW Formation

The only *Class II KNOX* gene that has recently been well characterized and extensively studied is *KNAT7* [12,13,14,15,16,30,31]. The role of KNAT7 TF as a regulator in SCW biosynthesis was first reported in *Arabidopsis* through the observation of the *irx* (irregular xylem) phenotype that occurred in a loss-of-function *knat7* mutant, *irx11* [30]. At the same time, the tight co-expression of the *KNAT7* TF gene along with SCW-specific *CesA* genes was reported using microarrays of *Arabidopsis* inflorescence stems undergoing SCW formation [11,32]. Promoter-GUS expression studies of *AtKNAT7* in *Arabidopsis* showed that it is highly expressed in developing xylem, phloem fibers, and cambium cells of inflorescence stems [13]. Wang et al. [15] recently examined whether several *Class II KNOX* genes from *Arabidopsis*, *KNAT3, KNAT4, KNAT5,* and *KNAT7*, were expressed during SCW deposition. All these *Class II KNOX* gene promoters regulated GUS expression in the vascular bundles in younger stems and intrafascicular fibers and vessel cells in older stems. These observations suggest that these *Class II KNOX* genes have similar expression patterns during the deposition of the SCWs. Qin et al. [16] also showed that while *KNAT7* expression was much higher in stem tissues, *KNAT3* expression remained similar in all tissues examined. Promoter-GUS fusions confirmed that *KNAT3* and *KNAT7* genes are co-expressed in developing xylem and interfascicular fibers in the *Arabidopsis* stem.

In poplar (*Populus balsamifera*), the expression of *PtKNAT7* gradually increases from the primary cell wall expansion stage to the mature xylem tissue formation stage, and from the youngest to the older internodes of stem [13]. Cotton *GhKNL1* was reported to be preferentially expressed in developing cotton fibers during SCW biosynthesis [33]. Switchgrass *KNAT7* also appears to be a functional ortholog of *Arabidopsis KNAT7*, based on its expression patterns [34]. In our laboratory, we studied the expression patterns of two *Class II KNOX* genes, *KNAT3* and *KNAT7*, in tobacco (*Nicotiana benthamiana*) [14]. Higher expression of *NbKNAT7* was seen in older stems of tobacco showing secondary growth followed by young stems and old leaves, while *NbKNAT3* displayed higher expression in older leaves followed by roots and young leaves. These two *Class II KNOX* genes were also found to be highly expressed during tension wood formation in aspen. The expression of *NbKNAT3* and *NbKNAT7* in young and old stems indicates that they play a role in wood formation. Thus, *Class II KNOX* genes are associated with SCW formation during xylem and fiber development.

### 1.3. Genetic Mutations in Class II KNOX Genes Further Clarify Their Role in SCW Formation

Until 2005, *KNAT7* was not often discussed in mutation studies of the *Class II KNOX* genes; however, a number of *Class II KNOX* mutations have recently been studied in detail (Table 1). A T-DNA insertion in the intron of the *KNAT7* gene resulted in a loss-of-function mutant, *irx11*, that showed only a moderately weak growth phenotype. The *irx11* mutant also exhibited the typical *irx* phenotype in xylem vessels that were collapsed due to weak SCW formation. The *irx11* mutant did not have significantly altered cellulose or xylan content compared to controls. No lignin content of these mutants was reported at that time. While discovering a set of novel TFs involved in SCW biosynthesis, Zhong et al. [12] associated *KNAT7* expression with SCW formation, and the dominant repression of *KNAT7* (DR-*KNAT7* mutants) affected SCW formation in both xylem and fiber cells (Table 1). Curiously, they did not observe the typical *irx* phenomenon in these DR-*KNAT7* mutants, a tell-tale sign of weak SCW formation; however, the cell wall thicknesses of both xylem vessels and fibers were reduced compared to controls (28% down in interfascicular fibers (IF), 26% down in vessels (V), and 80% down in xylary fibers (XF)). Several monosaccharides from the cell walls of DR-*KNAT7* mutants were reduced by 20–30%, except for arabinose, which was increased by 18%. The overexpression of *KNAT7* did not increase the SCW thickness of fibers and vessels. These results indicated that KNAT7 could be a positive regulator of SCW formation in *Arabidopsis*. However, Li et al. [13] reported a contrasting observation that loss-of-function mutants in the *AtKNAT7* gene resulted in differential thicknesses of interfascicular and xylary fibers compared to vessels (58% up in IF, 35% down in V, and 31% up in XF; Table 1). The vessels walls were thinner, resulting in collapsed xylem vessels that showed the *irx* phenotype (similar to [30]); however, the interfascicular fibers were significantly thicker than in the wild type control, suggesting that KNAT7 is a transcriptional repressor of fiber SCW formation (but a transcriptional activator of vessel SCW formation). *KNAT7* overexpression lines exhibited thinner fiber walls (57% down in IF) with normal vessel and xylary fiber cell walls. Interestingly, even though many SCW-specific cellulose and xylan synthesis genes were upregulated in these mutants, no quantitative changes in cellulose or xylan were reported. All ten lignin synthesis genes tested were upregulated along with an 11% increase in lignin content of cell walls from the stem. Li et al. [30] speculated that KNAT7 interacts with different partner proteins in different cell types to form functionally distinct complexes. Recently, the regulatory roles of other members of the *Class II KNOX* gene family, *KNAT3, KNAT4,* and *KNAT5*, in SCW formation were explored in *Arabidopsis* inflorescence stems [15,16] (Table 1). Loss-of-function mutants of *knat3, knat4,* and *knat5* did not produce any *irx* phenotype, as observed in the case of loss-of-function mutants of *knat7* [15]. This could be due to the functional redundancy of *KNOX II* genes. However, *knat3/knat7* double mutants displayed an enhanced *irx* phenotype compared to single *knat7* mutants. These double mutants had thinner interfascicular fiber cell walls compared to the single mutants and wild-type plants (40% down in IF) indicating a potentially positive regulatory role of KNAT3 in combination with KNAT7 in xylem SCW development. Even though many SCW genes were highly expressed in the *knat3/knat7* double mutants, the cellulose and xylan contents of their cell walls were reduced by 19% and 43%, respectively, and the changes in lignin content were not significant. The Syringyl to Guaicyl (S/G) lignin ratio was down by 83%; however, it was not possible to correlate all these cell wall content changes with the changes in gene expression patterns. In addition, the severe *irx* phenotype in these double mutants indicated the overlapping roles and partial functional redundancy of KNAT3 and KNAT7 in xylem vessel development during SCW formation. Furthermore, *KNAT3* overexpression in *Arabidopsis* resulted in thickened interfascicular fibers in the SCW of inflorescence stems [15]. This study described KNAT3 as a potential transcriptional activator, working together with KNAT7 to promote SCW biosynthesis in xylem vessels. A synergistic interaction of KNAT3 and KNAT7 to regulate monolignol biosynthesis in *Arabidopsis* was also reported in another study [16]. Most importantly, they attempted to link S-lignin formation with *KNAT3* and *KNAT7* expression; however, they could not show the direct transcriptional regulation of a key gene, ferulate 5-hydroxylase (*F5H*), involved in S-lignin formation by KNAT3 or KNAT7. Similar to the earlier observation by Wang et al. [15], the overexpression of KNAT3 also caused thickening in the interfascicular fiber walls, indicating the positive regulation of interfascicular fiber wall development by KNAT3. These studies by Wang et al. and Qin et al. [15,16] reconciled the paradoxical observations about *KNAT7* mutants in *Arabidopsis* and indicated that KNAT3 and KNAT7 might be working synergistically in fibers, but antagonistically in vessels, during the regulation of SCW biosynthesis (Table 1).

Similar observations regarding the function of KNAT7 in SCW formation were reported recently in rice (Table 1). Wang et al. [35] characterized the *KNAT7* transcription factor gene that controls SCW wall thickening in the stem. Interestingly, KNAT7 also regulates cell expansion in rice grains. An *Osknat7* CRISPR/CAS9 mutant had a thicker wall in fiber cells than that in the wild type, similar to the *Arabidopsis knat7* mutant, and transgenic rice plants overexpressing KNAT7 had opposite effects. Interestingly, the *Osknat7* mutant also exhibited a larger grain size due to cell expansion in spikelet bracts. The authors proposed that KNAT7 plays a negative regulatory role in SCW formation, similar to *Arabidopsis* [13]. The negative regulation of SCW formation by KNAT7 TF was also supported by Gong et al. [33] in cotton fibers (Table 1). The dominant repression of cotton KNAT7 (*GhKNL1*) resulted in abnormal fibers of shorter length in the cotton mutant compared to the controls, suggesting that cell elongation and SCW formation are also related to the function of KNAT7 in cotton. The overexpression of cotton KNAT7 in *Arabidopsis* produced thinner interfascicular fibers without any changes in the vessel or xylary fiber thickness. Thus, these *Class II KNOX* genes are involved in SCW formation in various plant species. 

### 1.4. Targeted Genetic Manipulations in Class II KNOX Genes Confirm Their Role in SCW Formation

Apart from the detailed study of *Class II KNOX* gene mutants, targeted genetic manipulations of *Class II KNOX* genes, especially, *KNAT7* genes have offered some additional clues regarding the functions of these genes (Table 2). While the overexpression of *KNAT7* in *Arabidopsis* did not produce any specific SCW phenotype [12], subsequently, Li et al. [13] reported that such experiments produced thin interfascicular fibers without any changes in wall thickness of vessels suggesting that KNAT7 TF is indeed a regulator of SCW formation. 

The successful complementation of *Arabidopsis knat7* mutants with the overexpression of the cotton *GhKNL1* gene [33] and poplar *PtKNAT7* [13] rescued the defective *irx* phenotype of the *knat7* mutants, suggesting the functional conservation of *KNAT7* genes among Arabidopsis, cotton, and poplar. The overexpression of cotton *GhKNL1* in *Arabidopsis* resulted in thinner interfascicular fibers and slightly thinner vessels walls without any change in the xylary fibers compared to control plants [33]. The overexpression of cotton *GhKNAT7* significantly reduced the deposition of lignocellulose in the interfascicular fibers of *Arabidopsis* [24]. However, the SCWs of the xylem fibers and vessels in the transgenic plants did not show any difference from the control plants. The dominant repression of the same cotton *KNAT7* orthologue in *Arabidopsis* produced thinner interfascicular fibers, but thicker vessels and xylary fiber walls, suggesting that KNAT7 can act as a negative or positive regulator of SCW formation in different cell types. 

In our laboratory, we generated *RNAi* lines of tobacco (*N. benthamiana*) that exhibited reduced expression of *KNAT7* [14]. *NbKNAT7* downregulated through a transient virus-induced gene silencing (VIGS) system resulted in increased xylem proliferation with thin-walled fiber cells. The glycome analyses of the cell walls showed increased polysaccharide extractability in 1 M KOH extracts of the VIGS-*NbKNAT7* lines, suggestive of SCW loosening. In addition, there were increased saccharification rates (40% higher than control) in stems of VIGS-*NbKNAT7* lines, which indicated the alteration of cell wall composition in VIGS lines downregulated for the *NbKNAT7* gene. Similar to the VIGS results, the stems of stable *RNAi* lines also showed increased xylem area in their stems as compared to control stems [14]. The cell walls of xylem fibers were thinner (over 50%) in the *RNAi* lines as compared to vector control lines. The stems of *KNAT7* repression lines in tobacco showed reduced expression of SCW genes that resulted in thinner fiber cell walls with altered cell wall composition [14]. All these results suggested that KNAT7 TF might act as a positive regulator of SCW formation in tobacco.

In a recent study performed in our laboratory by Ahlawat et al. [17], transgenic poplar plants overexpressing *PtKNAT7* and *AtKNAT7* genes showed enhanced expression of the SCW genes *CesA8, IRX9, PAL,* and *CCR,* and reduced expression of the same genes in the poplar *PtKNAT7* antisense plants. These results further suggested a positive regulatory role of KNAT7 in SCW formation in poplars. In addition, the genetic suppression of *KNAT7* in transgenic poplar stems reduced lignin content by about 6% and altered the lignin composition (S/G ratio) of poplar wood with increased saccharification ability (44–53% higher saccharification efficiency over control plants). Yoo et al. [36] also reported a negative correlation between lignin content and the saccharification efficiency of woody tissues and a positive correlation between the S/G ratio and the saccharification efficiency of SCW biomass. Therefore, a change in the S/G ratio and reduction in lignin content might be important for improving the saccharification efficiency of SCW biomass. All the studies reported so far in Arabidopsis and other higher plants suggest that KNAT7 acts differentially as a negative and positive regulator of SCW biosynthesis in different cell types of the same plant or in different plant species. 

### 1.5. Transcriptional Network of the Class II KNOX Genes Involved in SCW Formation

**Figure 2:**
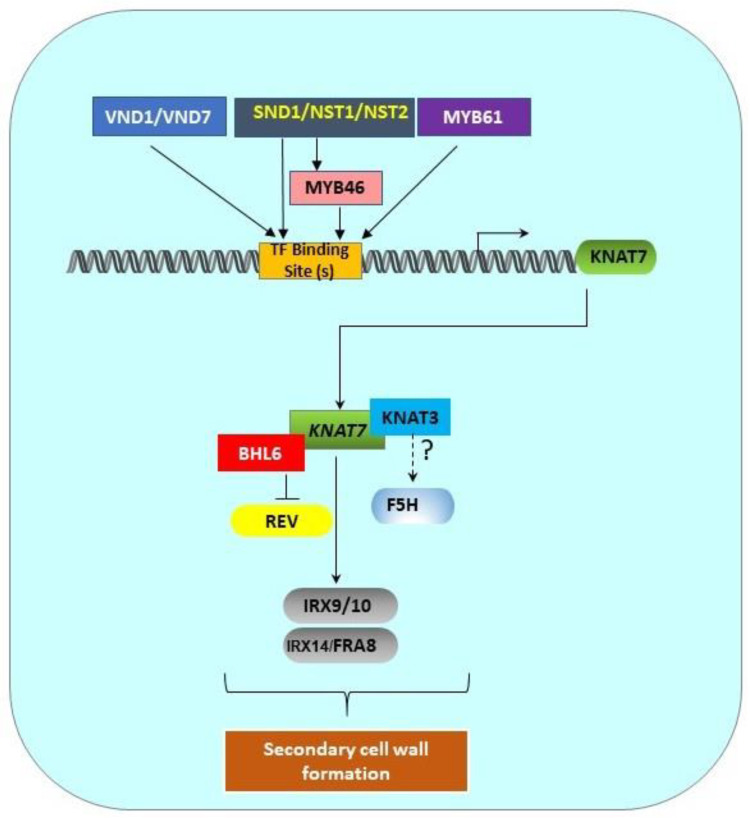
Transcriptional regulation pathway of *KNAT7* gene. SCW-associated upstream transcription factors (MYB61, SND1/NST1/NST2, VND1/VND7) and MYB46 directly bind the binding sites in the *KNAT7* gene promoter to regulate the expression of the *KNAT7* gene. KNAT7 positively regulates the expression of various xylan synthesis genes (*IRX9/10* and *IRX14L/FRA8*). Interactions between KNAT7 and KNAT3 TFs might regulate F5H expression, and the interactions between KNAT7 and BLH6 negatively regulate the expression of the homeodomain-ZIP (HD-ZIP) TF gene Revoluta. All these interactions ultimately regulate SCW formation in higher plants. All genes are shown as rounded rectangles and proteins are indicated by rectangles.

A complex network of transcription factors regulates SCW biosynthesis in plants [8,9,37,38,39,40]. Among these, some Class II KNOX TFs also regulate SCW biogenesis. The major constituents of the SCW are cellulose, lignin, and hemicelluloses [6]. Cellulose is a polymer of glucose synthesized at the plasma membrane by the cellulose synthase (CesA) complex [41], while lignin is composed of guaiacyl (G), syringyl (S), and p-hydroxyphenyl (H) units that are synthesized through the phenylpropanoid pathway [42]. Xylan is the major hemicellulose component in the SCW and consists of a linear backbone of β-(1–4)-linked D-xylosyl (Xyl) residues and α-linked (OMe(methyl)) glucuronic acid (GlcA) side branches [43]. Many specific genes involved in cellulose, hemicellulose, and lignin biosynthesis pathways have previously been identified in plants (e.g., [43,44,45]) and it was anticipated that Class II KNOX TF proteins might directly regulate the expression of some of these genes. The first direct evidence of KNAT7-mediated regulation of xylan biosynthesis in the SCW was reported only recently by He et al. [31], who demonstrated that KNAT7 physically binds to the promoters of the xylan biosynthetic genes, *IRREGULAR XYLEM 9* (*IRX9*)*, IRX10, IRX14L*, and *FRAGILE FIBER 8 (FRA8*; Figure 2). Wang et al. [46] also reported the involvement of KNAT7 in xylan synthesis during mucilage production. While various cellulose and lignin biosynthesis genes have been shown to be differentially expressed in various *knat7* mutants and during the ectopic expression of the *KNAT7* gene in transgenic plants, the direct regulation of any of these SCW genes by KNAT7 TF has not yet been reported. In addition, no information is currently available on transcriptional regulation by the TFs encoded by the other three *Class II KNOX* genes, namely *KNAT3*, *KNAT4*, and *KNAT5,* or their orthologs in any other plant species. 

### 1.6. Upstream Top- and Mid-Level Master Switches Control the Expression of KNAT7

The expression of KNAT7, a lower-level TF, is directly regulated by top and middle-level upstream TFs, such as NAC and MYB proteins [9,38]. SCW-associated NACs, such as SND1, NST1, NST2, VND6, and VND7, are top-level master switches that directly control KNAT7 expression [12] (Figure 2). Zhong et al. [47] reported that SND1, a master switch of SCW formation in *Arabidopsis* fibers, directly controls *KNAT7* expression. The downregulation of SND1 and its homolog NST1 also caused the downregulation of *KNAT7* expression [48]. Zhong et al. [12] identified the direct targets of SND1, and they discovered that the expressions of KNAT7, MYB46, MYB103, and SND3 are directly under the control of SND1. In addition, the SND1 homologs NST1, NST2, VND6, and VND7 were also found to directly activate KNAT7, among many other TFs. Zhong et al. [49] also identified 19-bp- secondary wall NAC binding elements (SNBEs); the *KNAT7* promoter has three such SNBEs at -616, -507, and -331 positions relative to the start codon. Interestingly, Zhong et al. [50] further showed that SND1 also directly regulates the expression of another mid-level master switch TF, MYB46 (Figure 2). Furthermore, a recombinant SND1 protein was able to bind to the *MYB46* promoter fragment and caused a mobility shift. Chromatin immunoprecipitation assays (ChIP) also enriched *MYB46* promoter fragments 3–5-fold, suggesting that SND1 directly binds to the *MYB46* promoter. Finally, the overexpression of MYB46 also upregulated *KNAT7*, and MYB46 appears to be a common target of secondary wall-associated SND1 homologs, including NST1, NST2, VND6, and VND7. Thus, in addition to SND1 and its homologs, MYB46 is also able to directly induce the expression of *KNAT7* (Figure 2). 

Zhong and Ye [50] showed that a 7-bp long consensus secondary wall MYB-responsive element (SMRE), ACC(A/T)A(A/C)(T/C) in *KNAT7* is directly involved in the MYB mediated activation of *KNAT7*, and the *KNAT7* promoter has three such SMREs located at the -802, -763, and -656 positions upstream of the translation initiation codon. Kim et al. [51] identified an eight bp motif that has one additional (T/C) before the SMRE and named it M46RE; *KNAT7* promoters have two such motifs. Ko et al. [52] comprehensively reviewed the literature regarding the functions of MYB46 and its close homolog, MYB83, and concluded that the expressions of many important genes involved in cellulose, hemicellulose, and lignin synthesis are directly regulated by MYB46/MYB83. Therefore, it appears that the defects in SCWs of the knat7 mutant might also be due to complex regulation by SND1 homologs and MYB46/83-regulated KNAT7 activities. 

KNAT7 functions as a common hub in several transcriptional networks that are involved in xylem differentiation and mucilage production, including the network that involves AtMYB61 [53]. The loss of *AtMYB61* function in a mutant resulted in defective xylem production, and it was shown that the MYB61 protein binds directly to an AC-rich element (ACC(A/T)A(A/C/T) in the promoter of *AtKNAT7*; there are three such AC-rich elements in the KNAT7 promoter at the -704, -665, and -558 positions upstream of the transcription start site. Interestingly, there are high similarities between these AC-rich elements, SMREs, and M46REs. Thus, MYB61 appears to be another upstream regulator of *KNAT7* in *Arabidopsis* (Figure 2).

### 1.7. Physical Interactions of Class II KNOX TF Proteins with Other Proteins

As homeodomain proteins, Class II KNOX proteins possess a DNA binding capacity, and many accessory proteins are known to physically interact with them (Table 3). Studies have reported that in at least four different plant species, namely Arabidopsis, rice, cotton, and poplars, the general scheme of interactions remains similar, while a number of species-specific variations have also been reported (Table 3).

In Arabidopsis, BEL1 encodes a TALE homeodomain-containing TF that heterodimerizes with KNOX proteins via interactions between the N-terminal region and the homeodomain and MEINOX domain of KNOX proteins [54]. Interestingly, no interactions were evident between BEL1 and KNAT3, KNAT4, and KNAT7 proteins; however, positive interactions were observed between BEL1 and KNAT5 and a few Class I KNOX proteins [54]. Furthermore, the C-terminal domain of BEL1, including the homeodomain, appears to be important for such specific interactions. However, Hackbush et al. [55] subsequently discovered that BEL1 and KNAT5 do not interact, but BLH (BELL-LIKE HOMEODOMAIN) proteins, such as BLH9/KNAT3 and BLH9/KNAT7, interact. In fact, out of four Class II KNOX proteins, eight BLH-proteins interacted with KNAT3, only BLH6 interacted with KNAT4, nine BLH proteins interacted with KNAT5, and two BLH proteins (BLH5 and BLH7) interacted with KNAT7. Another group of proteins that are known to interact with KNOX proteins are the ovate family proteins (OFPs) that are repressors of transcription and are involved in plant growth and development [55,56,57,58,59]. According to Hackbush et al. [55], five OFPs interact with KNAT3 and four different OFPs interact with KNAT4, KNAT5, and KNAT7. However, Li et al. [57] reported that only OFP4 showed strong Y2H interactions with KNAT7, while OFP1 showed only weak interactions. However, bimolecular fluorescence complementation (BiFC) and mutation data confirmed that KNAT7, OFP1, and OFP4 interact and play an important transcriptional repressor role during SCW formation (Table 3). Thus, interactions among BLH, OFPs, and KNOX proteins appear to play some major roles in plant development, including in SCW formation. Contrary to some of these findings, Liu et al. [60] showed that BLH6 specifically interacts with KNAT7, which represses commitment to SCW formation, and this interaction of TFs modulates the expression of the homeodomain-ZIP (HD-ZIP) TF gene, Revoluta (Figure 2). KNAT7 is a putative transcriptional repressor in *Arabidopsis* leaf protoplasts, and its repression is enhanced by physical interaction with OFP1 and OFP4 [57]. This was confirmed by the presence of *irx* vessels and altered fiber cell wall phenotypes displayed by *ofp4* single and *ofp4/ knat7* double mutants, similar to *knat7* single mutants. OFP1 and OFP4 are also components of the BLH6–KNAT7 multi-protein complex and may modulate the activity of the BLH6–KNAT7 complex [58]. KNAT7 also physically interacts and forms functional complexes with MYB75 and BLH6, which are involved in SCW formation [61,62] (Table 3). *blh6* knockout mutants displayed thicker cell walls in their interfascicular fibers, similar to *knat7* mutants [62], suggesting its role as a transcriptional repressor controlling SCW formation in interfascicular fibers through its interactions with KNAT7. 

*Arabidopsis* Class II KNOX TFs are also known to interact with each other. The Y2H data from Hackbush et al. [55] showed that KNAT3 physically interacts with KNAT4, KNAT4 interacts with KNAT7, and KNAT5 interacts with KNAT7. Recently, KNAT7 was reported to form a functional heteromeric complex with KNAT3 and regulate SCW formation, possibly via *F5H*, a syringyl lignin gene in *Arabidopsis* (Figure 2 and Table 3). Qin et al. [16] showed that a significant downregulation of the *F5H* gene, a key gene known to play a significant role in S-lignin formation, occurred in a *KNAT3*/*KNAT7* double mutant and as a result, the S/G lignin ratio was reduced by 83–84% compared to wild type stems. However, yeast-one-hybrid (Y1H) experiments did not show direct binding of either the KNAT7 or KNAT3 protein with the F5H gene promoter. They suggested that a larger complex (including KNAT3 and/or KNAT7) might regulate F5H gene expression in *Arabidopsis*. They also reported that KNAT3, but not KNAT7, can physically interact with the known top-level master regulators NST1 and NST2, but not SND1 (Table 3). However, neither NST1 nor NST2 alone or in combination with KNAT3 could activate the F5H promoter, suggesting that some other factors required for the formation of this complex were still missing in their experiments. In the poplar genome, there are two closely related F5H (or CAld5H) genes [63,64]. Wang et al. [64] discovered that 12 TFs are co-expressed with CAld5H genes. Only BLH6a and BLH6b are specifically bound to the *CAld5H2* promoter, and BLH2 is bound to both the promoters. BLH6 is also a transcriptional repressor. No mention of any KNOX II TFs in the BLH complex was made in this work. Bhargava et al. [65] showed that MYB75 and MYB5 both interact with KNAT7 (Table 3). 

Ma et al. [24] reported interactions among GhBEL1-like and GhKNOX II proteins from cotton (Table 3). GhBEL1, GhBLH1, and GhBLH6 interact with GhKNAT7. Moreover, GhKNAT7 interacts with GhMYB75, GhOFP1/5/4, and GhBLH1/5/6, forming heteromers. KNAT7 can form heterodimeric interactions (KNAT7–BLH and KNAT7–MYB) and at the same time can form trimeric interactions (KNAT7-BLH-OFP) to regulate SCW biosynthesis, and the functional conservation of these interactions in different plant species will help us to understand the complex regulatory network of SCW formation. Wang et al. [66] recently showed that the microtubule-associated GhIQD14 protein also interacts with the GhKNL1 protein (GhKNAT7) to regulate SCW formation; Arabidopsis and rice have similar genes encoding similar IQD14 proteins (Table 3).

MYB61 is one of the TFs that directly regulates the expression of KNAT7 in *Arabidopsis* [53], (Figure 2). In rice, a gibberellin-mediated DELLA-NAC signaling pathway regulates cellulose synthesis [67], and KNAT7, BELL, and OFP2 are known to interact during vasculature development [68]. NAC29/31directly regulates the expression of MYB61, which in turn activates *CesA* expression (Table 3). Wang et al. [35] recently showed that interactions between KNAT7 and NAC31 suppress the activation of MYB61 expression, suggesting that the order of signal transduction in SCW formation may have changed during the evolution of dicots and monocots [40]. Similarly, biochemical and gene expression studies in rice revealed that KNAT7 negatively regulates cellulose biosynthesis and cell expansion by interacting with NAC31 and a cell growth master regulator, growth regulating factor 4 (GRF4), which is known to control the expression of the expansin genes that regulate grain size (Table 3).

In a recent report, it was shown that KNAT7 and MYB6 heterodimers repressed SCW development in poplar and Arabidopsis while promoting anthocyanin synthesis [69]. The overexpression of MYB6 in transgenic poplar resulted in reduced SCW deposition, accompanied by the repressed expression of SCW biosynthetic genes. MYB6 has a DNA binding domain and interacts with the bHLH protein. KNAT7 also interacts with MYB6, MYB75, and MYB115 based on Y2H and BiFC data (Table 3). Therefore, it appears that the complex interactions of KNAT7 proteins with other cellular proteins play a major role in SCW formation in higher plants. 

## 2. Concluding Remarks and Future Perspectives

Recent studies have indicated that *Class II KNOX* genes are expressed during SCW formation in Arabidopsis and other higher plants. The expression of these genes in tissues undergoing SCW thickening and the effects of mutations in KNAT3 and KNAT7 genes on SCW synthesis clearly suggests their role in the transcriptional regulation of the genes involved in SCW formation. A clear understanding of the role of KNAT4 and KNAT5 in this process still awaits; if those genes are redundant in function, then their functions need to be ascertained using mutant complementation analysis with other *Class II KNOX* genes. Although this review focused on SCW formation, a few other metabolic processes in the life cycle of a plant, such as mucilage production, have been associated with *Class II KNOX* genes. As Romano et al. [53] suggested, KNAT7 appears to be a major hub where several pathways converge to coordinate multiple aspects of resource allocation in plants. 

Some ambiguity still exists around whether KNAT7 acts as a transcriptional activator or suppressor in SCW development. The suppression of KNAT7 function increased SCW formation in interfascicular fibers but resulted in reduced cell wall synthesis in xylary fibers with collapsed vessels, suggesting that it is a transcriptional suppressor [13,15]. Quite contrasting results were observed by other authors, who suggested that KNAT7 is a transcriptional activator [12,14,15,17,31,33]. Recent reports by Wang et al. and Qin et al. [15,16] reconciled these observations, suggesting that KNAT7 acts as a suppressor in interfascicular fibers but as an activator in vessels and xylary fibers. How the same TF plays these two contrasting roles is still unknown. These studies showed a differential regulatory role for KNAT7 depending on the tissue and cell type and its interacting partners. It is also possible that there are species-specific variations in KNAT7 function in SCW biosynthesis. More studies are required to answer the questions addressing the functional ambiguity of KNAT7 and how it interacts with cell wall TFs and other KNOX II proteins to regulate SCW formation. Furthermore, a detailed investigation into the regulatory network and downstream targets of Class II KNOX TF proteins is required to understand the transcriptional regulation of SCW formation. These studies will help us to modify cell wall formation in transgenic plants and enhance saccharification, as we recently showed [14,17]. Our understanding of the molecular controls of the deposition of each call wall component will help us to design cell walls for improved biomass production and reduced recalcitrance to bioconversion to ethanol. The modification of identified TFs through genetic engineering could help in overcoming some of the current bottlenecks leading to the realization of renewable bioenergy.

## Figures and Tables

**Table 1:** Gene Mutations in *Class II KNOX* genes and their effect on SCW formation.

Target Gene	Mutation	Type of Mutation	Anatomy of Mutants	References
*AtKNAT7*	*irx11*	T-DNA insertion	Irregular xylem with collapsed vessels.	[30]
*AtKNAT7*	-	Dominant repression	Reduced cell wall thickness of both xylem vessels and fibers; reduced composition of several monosaccharides from the cell walls.	[12]
*AtKNAT7*	*irx11*	Loss-of-function mutation	Thinner vessels walls resulted in a collapse of xylem vessels that showed the *irx* phenotype and thicker interfascicular fibers compared to controls; increase in lignin content.	[13]
*AtKNAT3, AtKNAT4, AtKNAT5*	Single mutants	T-DNA insertion	No *irx* phenotype.	[15]
*KNAT3/KNAT7*	Double mutant	T-DNA insertion	Enhanced irregular xylem (*irx*) phenotype characterized by weak inflorescence stem; reduced interfascicular fiber wall thickness and modified cell wall composition.	[15]
*KNAT3/KNAT7*	Double mutant	Chimeric repression	Thinner interfascicular fiber cell walls compared to single mutants and wild type (WT); reduced cellulose and xylan and reduced S/G lignin ratio.	[16]
*OsKNAT7*	*CRISPR/CAS9*	T-DNA insertion	Thicker fiber cell walls; larger grain size due to cell expansion in spikelet bracts.	[35]
*GhKNL1*	-	Dominant repression	Abnormal shorter fiber length.	[33]

**Table 2:** Genetic manipulation of *Class II KNOX* genes in different plant species.

Gene Used	Target Plant	Gene Modification Method	Impact on Transgenic Plants	References
*AtKNAT7*	Arabidopsis	Overexpression	Thin interfascicular fiber walls, but no change in vessel wall thickness.	[13]
*Cotton GhKNL1*	Arabidopsis	Overexpression	Thinner interfascicular fibers and slightly thinner vessel walls, but no change in xylary fibers.	[33]
*Cotton GhKNAT7*	Arabidopsis	Overexpression	Reduced deposition of lignocellulose in interfascicular fibers, but no change in the SCWs of xylem fibers and vessels.	[24]
*NbKNAT7*	Tobacco	Downregulation by VIGS and *RNAi*	Increased xylem proliferation with thin-walled fiber cells, increased polysaccharide extractability, and higher saccharification rate.	[14]
*AtKNAT7*	Arabidopsis	Dominant repression	Reduced expression of SCW genes that resulted in thinner fiber cell walls with altered cell wall composition.	[12]
*PtKNAT7*	Poplar	Overexpression	Enhanced expression of SCW genes, CesA8, IRX9, PAL, and CCR.	[17]
*PtKNAT7*	Poplar	Downregulation by antisense	Reduced expression of SCW genes, reduced lignin content, altered lignin composition (S/G ratio), and increased saccharification.	[17]

**Table 3:** Protein–protein interactions among Class II KNOX proteins and other TFs.

Species	Class II KNOX Proteins	Interacting Proteins	Biological Function	Reference
Arabidopsis	AtKNAT7	AtMYB75	SCW formation.	[61,65]
		AtMYB5	SCW formation.	[65]
		AtOFP1/4	KNAT7 transcriptional repression enhanced during SCW formation.	[57]
		AtBLHs	SCW formation	[55,60,62]
		AtKNAT3	Regulates S-lignin formation.	[55]
	AtKNAT3	NST1/2	Possibly regulates *F5H* gene expression to promote syringyl lignin synthesis.	[16]
		AtBLH1	SCW formation.	[55]
		AtKNAT7	Possibly regulates S-lignin formation.	[16]
Cotton	GhKNAT7	GhMYB75	SCW biosynthesis.	[24]
		GhBLH1/5/6	SCW biosynthesis.	[24]
		GhBEL1	SCW biosynthesis.	[24]
		GHOFP1/5/4	SCW biosynthesis.	[24]
		GhIQD14	SCW biosynthesis.	[66]
Poplar	PtKNAT7	PtMYB6	Promotes anthocyanin synthesis and represses SCW development.	[69]
		PtMYB75	SCW formation.	[69]
		PtMYB115	SCW formation.	[69]
Rice	OsKNAT7	OsGRF4	Negatively regulates cellulose biosynthesis and cell expansion.	[35]
		OsOFP2	Vasculature development.	[68]
		OsNAC29/31	Suppresses the activation of MYB61 expression during SCW formation.	[35]

## Abbreviations

BLH	BEL-like homeodomain
BiFc	Bimolecular fluorescence complementation
CesA	Cellulose synthase
ChIP	Chromatin immunoprecipitation assays
F5H	Ferulate 5-hydroxylase
HD	Homeodomain
irx	Irregular xylem
KNOX	Knotted-like homeobox
OFPs	Ovate family proteins
SCW	Secondary cell wall
S/G	Syringyl to Guaicyl lignin ratio
TALE	Three amino acid loop extension
TFs	Transcription factors
VIGS	Virus-induced gene silencing
Y1H	Yeast-one hybrid
Y2H	Yeast-two hybrid
